# Habitat radiomics and deep learning fusion nomogram to predict EGFR mutation status in stage I non-small cell lung cancer: a multicenter study

**DOI:** 10.1038/s41598-024-66751-1

**Published:** 2024-07-10

**Authors:** Jingran Wu, Hao Meng, Lin Zhou, Meiling Wang, Shanxiu Jin, Hongjuan Ji, Bona Liu, Peng Jin, Cheng Du

**Affiliations:** 1 Department of Oncology, General Hospital of Northern Theater Command, Shenyang, 110840 China; 2 Department of Thoracic Surgery, General Hospital of Northern Theater Command, Shenyang, 110840 China; 3 Department of Thoracic Surgery, Yuebei People’s Hospital Affiliated to Shantou University Medical College, Shaoguan, 512025 China; 4 Department of Oncology, The Second Affiliated Hospital of Shandong First Medical University, Taian, 271000 China

**Keywords:** Cancer imaging, Non-small-cell lung cancer

## Abstract

Develop a radiomics nomogram that integrates deep learning, radiomics, and clinical variables to predict epidermal growth factor receptor (EGFR) mutation status in patients with stage I non-small cell lung cancer (NSCLC). We retrospectively included 438 patients who underwent curative surgery and completed driver-gene mutation tests for stage I NSCLC from four academic medical centers. Predictive models were established by extracting and analyzing radiomic features in intratumoral, peritumoral, and habitat regions of CT images to identify EGFR mutation status in stage I NSCLC. Additionally, three deep learning models based on the intratumoral region were constructed. A nomogram was developed by integrating representative radiomic signatures, deep learning, and clinical features. Model performance was assessed by calculating the area under the receiver operating characteristic (ROC) curve. The established habitat radiomics features demonstrated encouraging performance in discriminating between EGFR mutant and wild-type, with predictive ability superior to other single models (AUC 0.886, 0.812, and 0.790 for the training, validation, and external test sets, respectively). The radiomics-based nomogram exhibited excellent performance, achieving the highest AUC values of 0.917, 0.837, and 0.809 in the training, validation, and external test sets, respectively. Decision curve analysis (DCA) indicated that the nomogram provided a higher net benefit than other radiomics models, offering valuable information for treatment.

## Introduction

Lung cancer ranks as the leading cause of cancer-related deaths globally, with non-small-cell lung cancer (NSCLC) constituting more than 85% of documented cases^1,2^. Precision medicine advancements, particularly targeted therapeutics based on driver gene analysis, have significantly prolonged the survival of NSCLC over the past two decades^3^. Among the frequent driver mutations in NSCLC, the Epidermal Growth Factor Receptor (EGFR) mutation stands out. Targeted therapies, such as Tyrosine Kinase Inhibitors (TKI) directed at EGFR, have notably improved the 5-year overall survival rate in advanced NSCLC to 88%. In the adjuvant therapy setting, EGFR-TKIs have been extensively employed in stage IB to IIIA NSCLC, substantially reducing the risk of recurrence and metastasis^4^. A retrospective cohort study^5^ revealed that adjuvant EGFR-TKIs post-surgical resection provided a sustained and clinically significant 5-year Disease-Free Survival (DFS) benefit in stage I NSCLC patients, both in stage IA (EGFR-TKIs vs. observation = 100.0% vs. 84.5%; P = 0.007) and stage IB (EGFR-TKIs vs. observation = 98.8% vs. 75.3%; P = 0.008). Neoadjuvant targeted therapy has proven effective and well-tolerated in patients with EGFR-positive early-stage NSCLC^6^. However, challenges persist in certain circumstances for stage I NSCLC patients, such as elderly individuals declining surgery and biopsy or those with high-risk factors for ground-glass opacity (GGO) undergoing cautious monitoring.

In clinical practice, the detection of EGFR mutations in tumor tissues primarily relies on surgical or biopsy specimens. However, this approach has limitations: (1) Invasive methods can lead to complications such as pneumothorax and hemoptysis^7^. (2) Tissue samples often represent only a fraction of a typically heterogeneous lesion, limiting their ability to fully characterize the lesion^8^. (3) Performing biopsies on stage I patients with relatively small tissues is challenging, and the limited quantity or quality of samples hampers the feasibility of conducting EGFR mutation testing. While circulating tumor DNA (ctDNA) in plasma has been utilized to detect EGFR mutations in NSCLC patients, the concordance rates between ctDNA and tumor tissues exhibit significant variation^8^. Moreover, ctDNA levels are relatively low in early-stage NSCLC, leading to low sensitivity and false-negative outcomes^9,10^. Therefore, there is an urgent need to develop a non-invasive and user-friendly model to predict EGFR mutations in stage I NSCLC.

The radiomics approach involves the conversion of medical images into quantitative data to assist noninvasive clinical decision-making^11^. Numerous studies have already demonstrated the efficacy of various radiomics or deep learning models in predicting EGFR mutations non-invasively^12–15^. The term “habitat” is used to describe distinct, regional, and heterogeneous volumes within a tumor, and habitat imaging involves obtaining these volumes^16^. Scholars have started incorporating habitat imaging into the field of radiomics, showcasing its superior performance compared to other methods^17^. The objective of this study was to investigate which CT-based radiomic model is more advantageous in predicting EGFR mutations in patients with stage I NSCLC. We developed, compared, and validated multiple CT-based models for identifying EGFR mutation status in stage I NSCLC patients, including intratumoral, peritumoral, and habitat region radiomics, as well as deep learning models. Finally, we constructed a nomogram by integrating clinical features with CT-based signatures, aiming to enhance its clinical applicability.

## Materials and methods

### Study design

**Figure 1:**
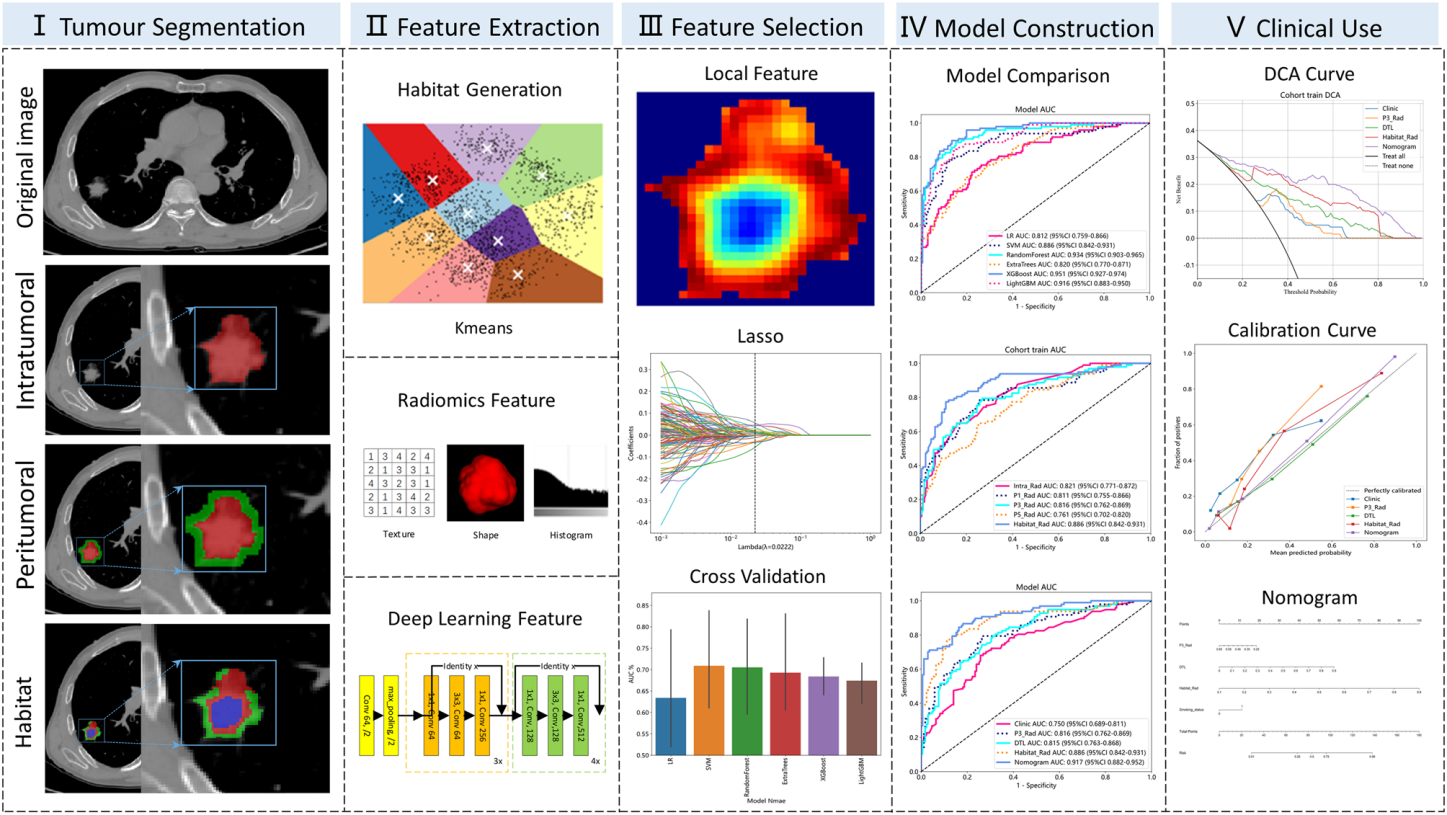
Overall workflow of this study.

Our study introduces four radiomic models encompassing intratumoral, peritumoral, and habitat region radiomics, along with deep learning models. The workflow of the study is illustrated in Fig. 1.

### Patients

**Figure 2:**
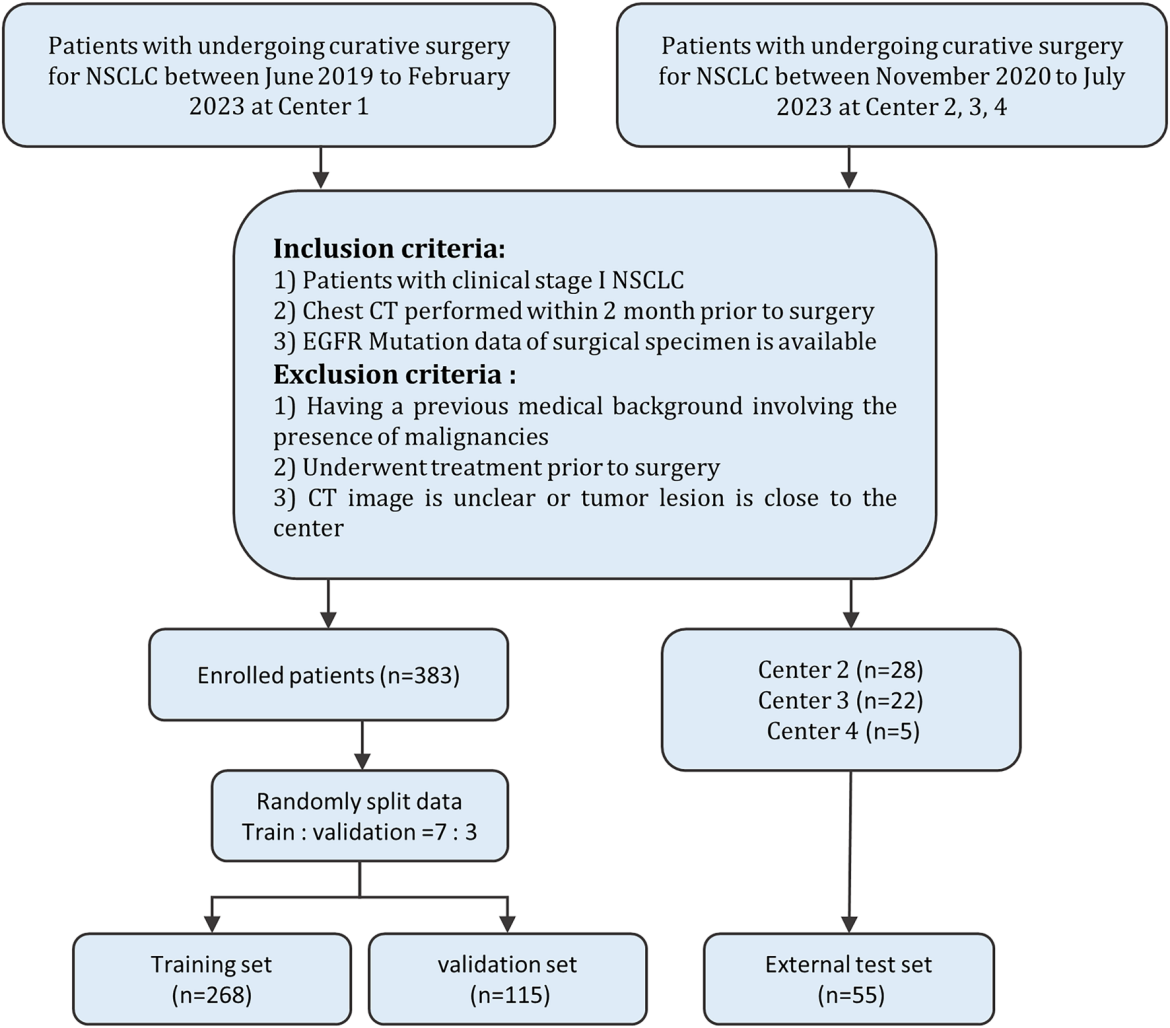
Flow chart of the patient recruitment pathway. Center 1, General Hospital of Northern Theater Command; Center 2, Yuebei People’s Hospital Affiliated to Shantou University Medical College; Center 3, Shandong First Medical University; Center 4, Shengjing Hospital of China Medical University.

We retrospectively enrolled patients with stage I NSCLC who underwent curative surgery from four academic medical centers. Preoperative non-enhanced CT images and clinical data were collected. Inclusion criteria: (1) Patients with clinical stage I NSCLC; (2) Chest CT performed within 2 months prior to surgery; (3) EGFR Mutation data of surgical specimen is available. The exclusion criteria were as follows: (1) with a history of other malignant tumors; (2) with therapy before surgery; (3) CT image is unclear or tumor lesion is close to the center. A total of 438 patients were included in this study (Fig. 2). Patients from center 1 were randomly split into a training set (n = 268) and a validation set (n = 115), while patients from centers 2, 3, and 4 formed the external test set (n = 55). EGFR mutations were determined using Next-generation sequencing (NGS) or amplification refractory mutation system (ARMS) methods. Baseline clinical and demographic data, including age, gender, pathological stage, smoking history, CT pattern, histopathological subtype, tumor location, and EGFR mutation status, were derived from medical records. This study was conducted according to the principles of the Declaration of Helsinki and approved by the Ethics Committee of the General Hospital of Northern Theater Command.

### Image acquisition, segmentation, and preprocessing

The ITK-SNAP 3.8.0 software (http://www.itksnap.org) was used to establish the region of interest (ROI). A stable pulmonary window (window width 1500 HU, window position − 500 HU) was employed, and an oncologist physician identified the target nodule, modifying the ROI boundary layer by layer without prior knowledge of the patient's clinical data and mutational status.

Due to the use of different CT scans in the present study, image preprocessing prior to segmentation and feature extraction was performed to make the radiomic features more robust and more suitable for further analysis. To standardize different CT images, two steps were applied: (1) Limiting the intensities of pixel values to the range of − 800 to 800 to mitigate the influence of extreme values and outliers. (2) Addressing voxel spacing inconsistencies in various volumes of interest (VOI) using the fixed resolution resampling method for spatial normalization, achieving a uniform voxel spacing of $1\;{\text{mm}} \times 1\;{\text{mm}} \times 1\;{\text{mm}}$.

### Peritumoral regions dilation and habitat generation

**Figure 3:**
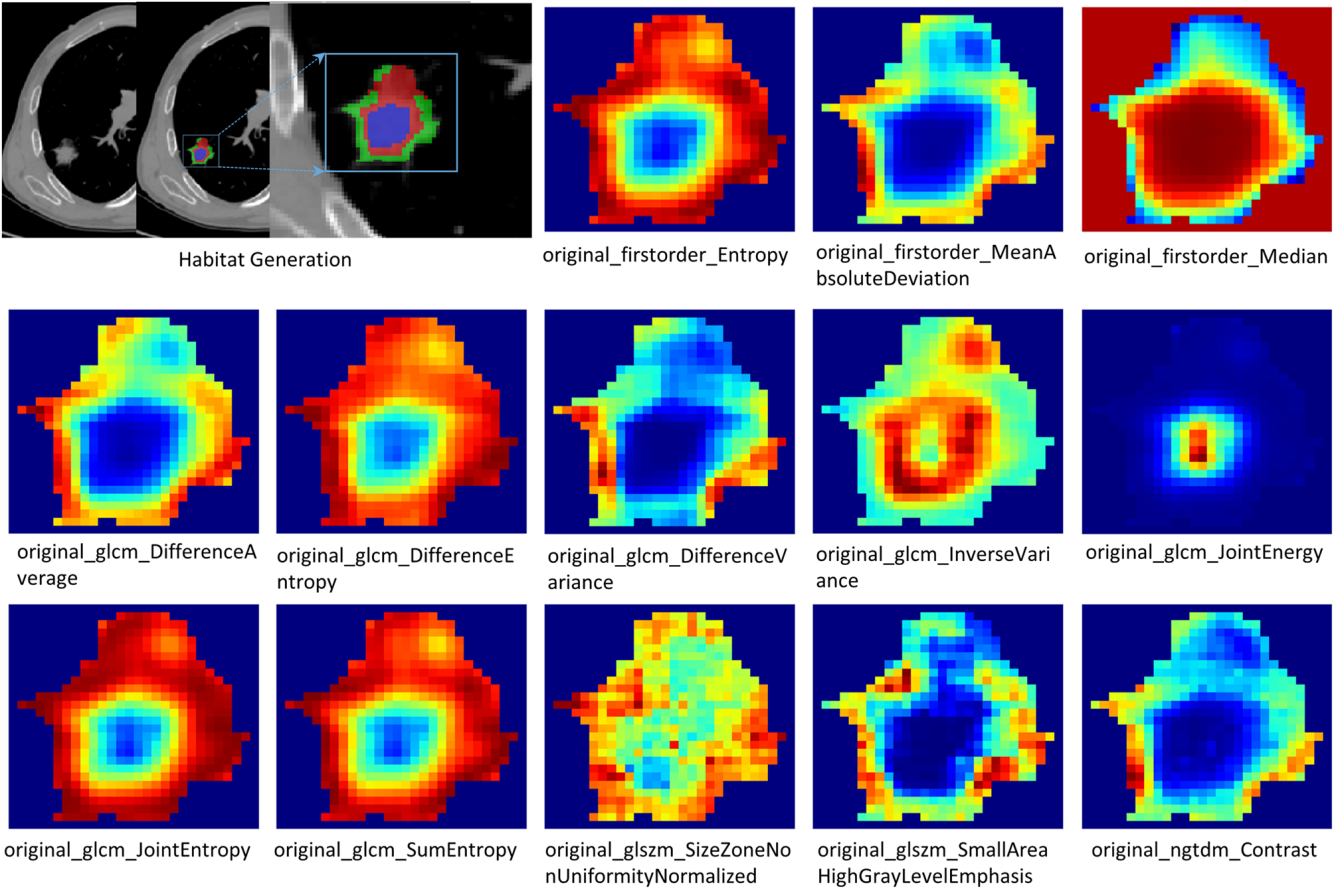
The generated habitat regions and 13 characteristics are presented.

The original Region of Interest (ROI) mask was systematically extended using the morphological dilation operator at varying radial distances. Different peritumoral regions were explored by configuring dilation intervals of 1 mm, 3 mm, and 5 mm to assess their impact on the predictive capabilities of the model. Local features, such as local entropy and energy values, were obtained by analyzing each voxel within the designated Volume of Interest (VOI). A moving window of size 3 × 3 × 3 was used to calculate the local features for every voxel, extracting 13 feature vectors per voxel. The K-means method was then applied to cluster sub-regions, resulting in the segmentation of the VOI into three distinct regions for each sample. Habitat generation and specific features were detailed in Fig. 3. Details are in the Supplementary Data 1.

### Feature extraction

Handcrafted features utilized in this study were categorized into three groups: (I) geometry, (II) intensity, and (III) texture. Specifically, 14 shape features were included. Additionally, we performed image transformations for feature extraction, with 18 first-order intensity features and 75 texture features for each transformation. The transformations included Wavelet, LoG, and 18 other methods, totaling 20 transformations. All features were extracted using the Pyradiomics tool (http://pyradiomics.readthedocs.io), adhering to feature definitions outlined by the Imaging Biomarker Standardization Initiative (IBSI)^18^.

### Feature selection

Test–retest and inter-rater analyses were conducted to ensure selected features were not influenced by segmentation uncertainties. Highly repeatable features with an ICC ≥ 0.85 were considered robust against segmentation uncertainties. Standardization using Z-scores ensured a normal distribution. P values for imaging features were calculated using a *t*-test, retaining features with a P-value < 0.05. Pearson's correlation coefficient was used to filter highly correlated features, implementing a greedy recursive deletion strategy. The minimum Redundancy Maximum Relevance (mRMR) algorithm was employed to mitigate overfitting.

### Radiomic models development

Machine learning models, including multi-layer perception (MLP), random forest (RF), support vector machine (SVM), logistic regression (LR), extreme gradient boosting (XGBoost), light gradient boosting machine (LightGBM), and extremely randomized trees (Extra-Trees), were applied to derive the intratumoral, peritumoral, and habitat regions radiomics signature from the final features. Optimized hyperparameters for each machine learning model are provided in Supplementary Data 2.

### Deep learning model development and model interpretability

Three classic transfer learning models (ResNet18, ResNet50, ResNet101) were evaluated in this study. The Deep Transfer Learning (DTL) signature was obtained for each sample using a deep learning model pre-trained on the ILSVRC-2012 dataset. The CT slice showing the maximum tumor ROI area was chosen as the original image and the gray values of the selected slice were then normalized using min–max transformation to ensure a range of [− 1, 1]. Subsequently, the cropped subregion image was resized to dimensions of 224 × 224 through the implementation of nearest interpolation. The learning rate employed in experiments was determined using the cosine decay learning rate algorithm. The specific learning rate used in our experiments is presented as follows:

$$\eta_{t}^{task - spec} = \eta_{min}^{i} + \frac{1}{2}\left( {\eta_{max}^{i} - \eta_{min}^{i} } \right)\left( {1 + \cos \left( {\frac{{T_{cur} }}{{T_{i} }}\pi } \right)} \right)$$

The minimum learning rate, denoted as $\eta_{min}^{i}$, is set to 0, while the maximum learning rate, denoted as $\eta_{max}^{i}$, is set to 0.01. The parameter $T_{i}$ represents the number of iteration epochs. Since the backbone part of the model utilizes pre-trained parameters, we perform fine-tuning on the backbone part at $T_{cur} = \frac{1}{2}T_{i}$ to ensure effective transfer of knowledge. Consequently, the learning rate for the backbone part is determined as follows:

$$\eta_{t}^{backbone} = \left\{ {\begin{array}{*{20}l} 0 \hfill & { \quad {\text{if}}\; T_{cur} \le \frac{1}{2}T_{i} } \hfill \\ {\eta_{min}^{i} + \frac{1}{2}\left( {\eta_{max}^{i} - \eta_{min}^{i} } \right)\left( {1 + \cos \left( {\frac{{T_{cur} }}{{T_{i} }}\pi } \right)} \right)} \hfill & { \quad {\text{if}} \;T_{cur} > \frac{1}{2}T_{i} } \hfill \\ \end{array} } \right.$$

The stochastic gradient descent (SGD) optimizer was employed to update the model parameters.

To enhance the interpretability of the Deep Learning Radiomics (DLR) model, Gradient-weighted Class Activation Mapping (Grad-CAM) was utilized for visualization. From Supplementary Fig. S3, it can be seen that the network with the attention mechanism can more precisely focus on information-rich lesion and border regions, regardless of wild-type or mutant status.

### Clinical signature and nomogram construction

Univariable and stepwise multivariable analyses were conducted on all clinical features. Due to the limited number of features, all clinical features were incorporated into the clinical model during its construction. The clinical model employed several of the same machine learning algorithms used in intratumoral radiomics. By amalgamating clinical features, peritumoral, habitat, and Deep Transfer Learning (DTL) signatures, a nomogram was formulated.

### Statistical analysis

We employed the independent sample *t*-test and the χ^2^ test to compare the clinical characteristics of the patients. The χ^2^ test was utilized for discrete variables, while the *t*-test was used for continuous variables involving only two groups. In the training cohort, we performed fivefold cross-validation and employed the Grid-Search algorithm to determine optimal hyperparameters and enhance the algorithm's performance.

The diagnostic performance was assessed using receiver operating characteristic (ROC) curves. Differences in AUC values between models were compared using the Delong test. The goodness of fit of the model was evaluated by the calibration curve and the Hosmer–Lemeshow test. Decision curve analysis (DCA) was conducted to appraise the clinical utility of the predictive models. All hypothesis tests were two-sided, and P < 0.05 indicated a significant difference.

### Ethical statement

The Institutional Review Board of General Hospital of Northern Theater Command approved this study. Further, informed consent from all participants was waived by the IRB because of the retrospective nature of this study.

## Results

### Clinical features of patients

The clinical features of enrolled patients are presented in Table 1. In our study, the mutation rates of EGFR were found to be 63.8%, 69.6%, and 70.9% in the training, validation, and test cohorts, respectively. EGFR mutation occurrence was higher in demographic groups characterized by female gender, non-smoking history, adenocarcinoma subtype, and the presence of ground glass nodules. Univariate and multifactorial analyses of clinical features in the training set were conducted, and odds ratios (OR) along with the corresponding P-values for each feature were computed (Table 2). Univariate analysis revealed that gender and smoking history were significantly different between the EGFR mutant and wild-type groups. Multivariate analysis revealed that smoking history (odds ratio (OR), 1.238; 95% confidence interval (CI), 1.087–1.412; P = 0.008) was independently correlated with the EGFR mutation status.

**Table 1:** Baseline demographic and clinical characteristics of the patients.

Characteristic	Training set (n = 268)		*P*	Validation set (n = 115)		*P*	External test set (n = 55)		*P*
	EGFR (+)	EGFR (−)		EGFR (+)	EGFR (−)		EGFR (+)	EGFR (−)	
Counts	171 (63.8)	97 (36.2)		80 (69.6)	35 (30.4)		39 (70.9)	16 (29.1)	
Age	60.7 ± 8.6	59.6 ± 9.8	0.355	59.6 ± 7.6	59.3 ± 9.2	0.837	60.2 ± 9.8	58.5 ± 8.3	0.539
Gender			0.001			0.351			0.091
Male	59 (34.5)	54 (55.7)		32 (40.0)	18 (51.4)		13 (33.3)	10 (62.5)	
Female	112 (65.5)	43 (44.3)		48 (60.0)	17 (48.6)		26 (66.7)	6 (37.5)	
Stage			0.450			0.102			0.412
AIS^a^	9 (5.26)	9 (9.3)		4 (5.0)	6 (17.1)		2 (5.1)	1 (6.3)	
IA	144 (84.2)	78 (80.4)		67 (83.8)	26 (74.3)		33 (84.6)	15 (93.8)	
IB	18 (10.5)	10 (10.3)		9 (11.3)	3 (8.6)		4 (10.3)	0	
Smoking history			< 0.001			0.121			0.067
No	132 (77.2)	51 (52.6)		59 (73.8)	20 (57.1)		36 (92.3)	11 (68.8)	
Yes	39 (22.8)	46 (47.4)		21 (26.3)	15 (42.9)		3 (7.7)	5 (31.3)	
CT pattern			0.005			< 0.001			0.655
Ground glass	85 (49.7)	55 (56.7)		45 (56.3)	14 (40.0)		12 (30.8)	7 (43.8)	
Mixed	54 (31.6)	14 (14.4)		27 (33.8)	7 (20.0)		15 (38.5)	5 (31.3)	
Solid	32 (18.7)	28 (28.9)		8 (10.0)	14 (40.0)		12 (30.8)	4 (25.0)	
Histological type			< 0.001			0.007			1.000
Adenocarcinoma	164 (95.9)	69 (71.1)		77 (96.3)	29 (82.9)		39 (100.0)	16 (100.0)	
Other	0	13 (13.4)		0	4 (11.4)		0	0	
Unknown	7 (4.1)	15 (15.5)		3 (3.8)	2 (5.7)		0	0	
Tumor location			0.461			0.768			0.940
Left upper lobe	44 (25.7)	21 (21.7)		19 (23.8)	9 (25.7)		7 (18.0)	2 (12.5)	
Left lower lobe	16 (9.4)	16 (16.5)		13 (16.3)	9 (25.7)		3 (7.7)	2 (12.5)	
Right upper lobe	68 (39.8)	34 (35.1)		32 (40.0)	12 (34.3)		18 (46.2)	8 (50.0)	
Right middle lobe	8 (4.7)	4 (4.1)		3 (3.8)	1 (2.9)		4 (10.3)	2 (12.5)	
Right lower lobe	35 (20.5)	22 (22.7)		13 (16.3)	4 (11.4)		7 (18.0)	2 (12.5)	

*Data was presented as mean ± SD, or n (%), unless otherwise stated. *AIS* adenocarcinoma in situ.

**Table 2:** Univariable and multivariable analysis of clinical features.

Characteristic	Univariate analysis		Multivariable analysis	
	OR (95% CI)	*P*	OR (95% CI)	*P*
Age	0.997 (0.992–1.002)	0.355	NA	
Gender	0.818 (0.743–0.901)	0.001	0.930 (0.822–1.052)	0.333
Stage	0.944 (0.839–1.062)	0.422	NA	
Smoking history	1.300 (1.175–1.439)	0.000	1.238 (1.087–1.412)	0.008
CT pattern	1.011 (0.952–1.074)	0.760	NA	
Histological type	1.037 (0.906–1.186)	0.658	NA	
Tumor location	1.006 (0.971–1.041)	0.785	NA	

### Performance of intratumoral, peritumoral, and habitat radiomics models

A total of 1834 handcrafted radiomic features in different subsets were extracted and further selected using the Lasso approach. The proportion of the coefficients of the selected features is shown in Supplementary Fig. S1. After feature selection, a fivefold cross-validation approach was employed to determine the most optimal machine learning technique for the development of a radiomic model. Selecting the model with the highest AUC on the external test set indicates the best machine learning model. The optimal machine learning algorithms used for the intratumoral, peritumoral 1 mm, peritumoral 3 mm, peritumoral 5 mm, and habitat regions were LightGBM, SVM, Extra-Trees, RF, and SVM, respectively. ROC curves for different machine learning methods were compared using the external test set. Details are shown in Supplementary Fig. S2.

**Figure 4:**
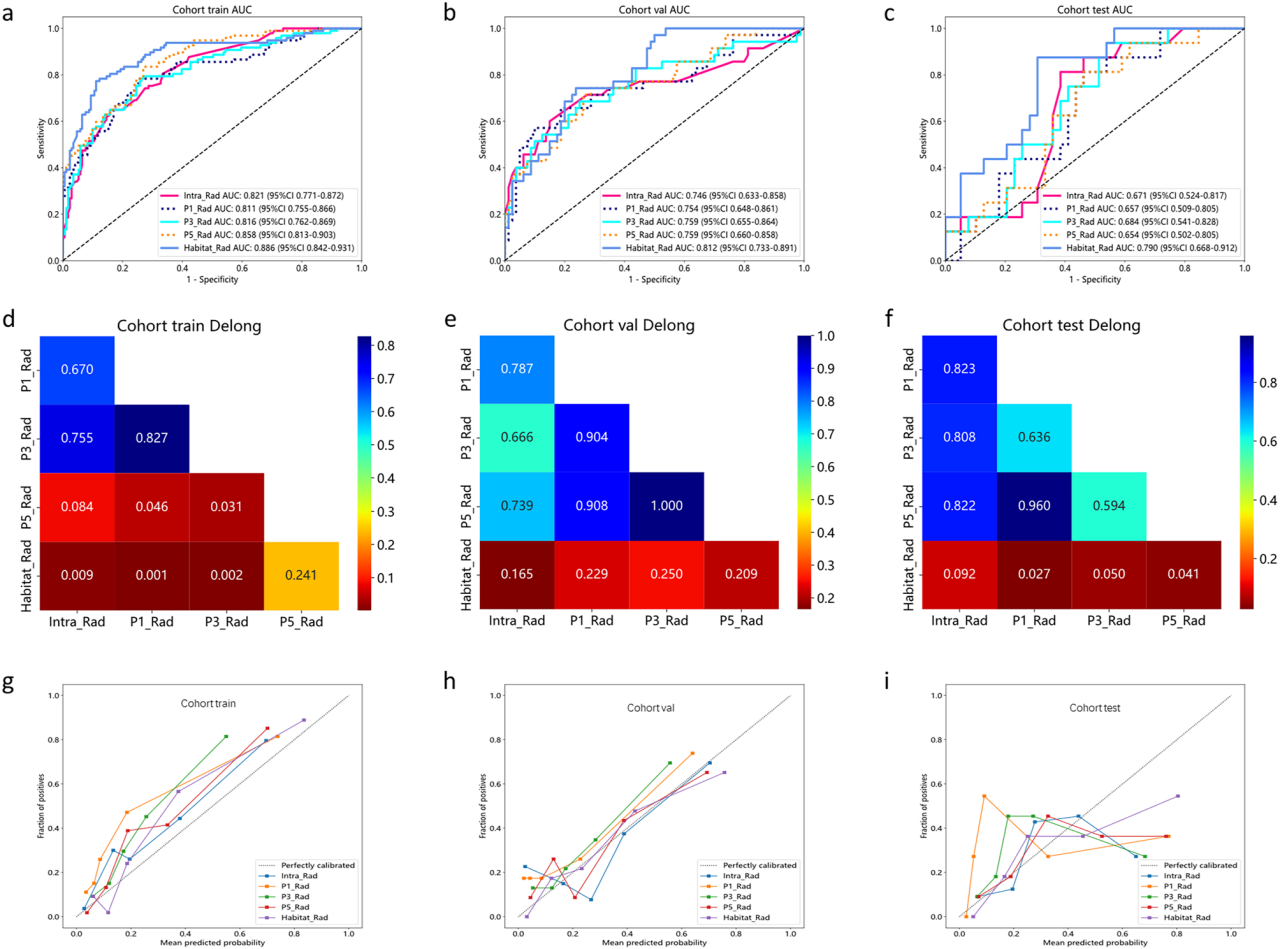
Receiver operating characteristic (ROC) curve of different models in the (**a**) train, (**b**) validation, (**c**) external test sets, respectively. Delong test of different models in the (**d**) train, (**e**) validation, (**f**) external test sets, respectively. Calibration curve of different models in the (**g**) train, (**h**) validation, (**i**) external test sets, respectively. Intra_Rad, intratumoral radiomics signature; P1_Rad, Peritumoral 1 mm radiomics signature; P3_Rad, Peritumoral 3 mm radiomics signature; P5_Rad, Peritumoral 5 mm radiomics signature; Habitat_Rad, habitat radiomics signature.

In the train cohort, the Habitat_Rad signature demonstrated the highest AUC (Area Under the Curve) of 0.886 (95% CI: 0.842–0.931). The Intra_Rad signature also showed a good AUC value of 0.821 (95% CI: 0.771–0.872). The AUC values for three different settings in the peritumoral regions were 0.811 (95% CI: 0.755–0.866), 0.816 (95% CI: 0.762–0.870), and 0.858 (95% CI: 0.813–0.903), respectively. In the validation cohort, the Habitat_Rad signature again showed the highest AUC (0.812, 95% CI: 0.733–0.891). In the external test cohort, the Habitat_Rad signature achieved the highest AUC (0.790, 95% CI: 0.668–0.912). The AUC value of the P3_Rad signature was 0.684 (95% CI: 0.541–0.828), which outperformed the other three radiomic signatures (Intra_Rad, 0.671; P1_Rad, 0.657; P5_Rad, 0.654). The accuracy, sensitivity, specificity, negative predictive value, and positive predictive value were listed in Supplementary Table S1. The Delong test was utilized to compare the AUC of different models (Fig. 4). Comparisons with P1_Rad, P3_Rad, and P5_Rad showed that the habitat exhibited a significant improvement in the external test cohort (P value < 0.05).

### Performance of the deep learning model

We employed three classic transfer learning models (ResNet18, ResNet50, ResNet101) in intratumoral regions to identify EGFR mutation status in stage I NSCLC. The AUC for the ResNet18 model was 0.710 (95% CI: 0.5498–0.8700) in the external test cohort, outperforming the ResNet101 and ResNet50 models (Table 3). In order to enhance the transparency of the model's decision-making process and explore its interpretability, gradient-weighted class activation mapping (Grad-CAM) was employed to provide visual representations of the model (Supplementary Fig. S3).

**Table 3:** The performance comparison of deep learning different models.

Model		AUC (95% CI)	Accuracy	Sensitivity	Specificity	PPV	NPV
resnet101	Train	0.717 (0.655–0.780)	0.675	0.635	0.753	0.818	0.537
	Validation	0.689 (0.582–0.797)	0.696	0.750	0.571	0.800	0.500
	Test	0.671 (0.506–0.836)	0.764	0.868	0.562	0.825	0.600
resnet18	Train	0.815 (0.763–0.868)	0.728	0.690	0.794	0.855	0.592
	Validation	0.713 (0.607–0.820)	0.696	0.662	0.771	0.869	0.500
	Test	0.710 (0.550–0.870)	0.745	0.789	0.688	0.857	0.550
resnet50	Train	0.862 (0.819–0.905)	0.776	0.743	0.835	0.888	0.648
	Validation	0.729 (0.628–0.830)	0.757	0.875	0.486	0.795	0.630
	Test	0.624 (0.474–0.775)	0.618	0.553	0.812	0.875	0.419

*PPV* positive predictive value, *NPV* negative predictive value.

### Clinical model and nomogram

All clinical information was used to construct a clinical model. The optimal machine learning algorithm for constructing clinical models is Extra-Trees (Supplement Fig. S2).

**Figure 5:**
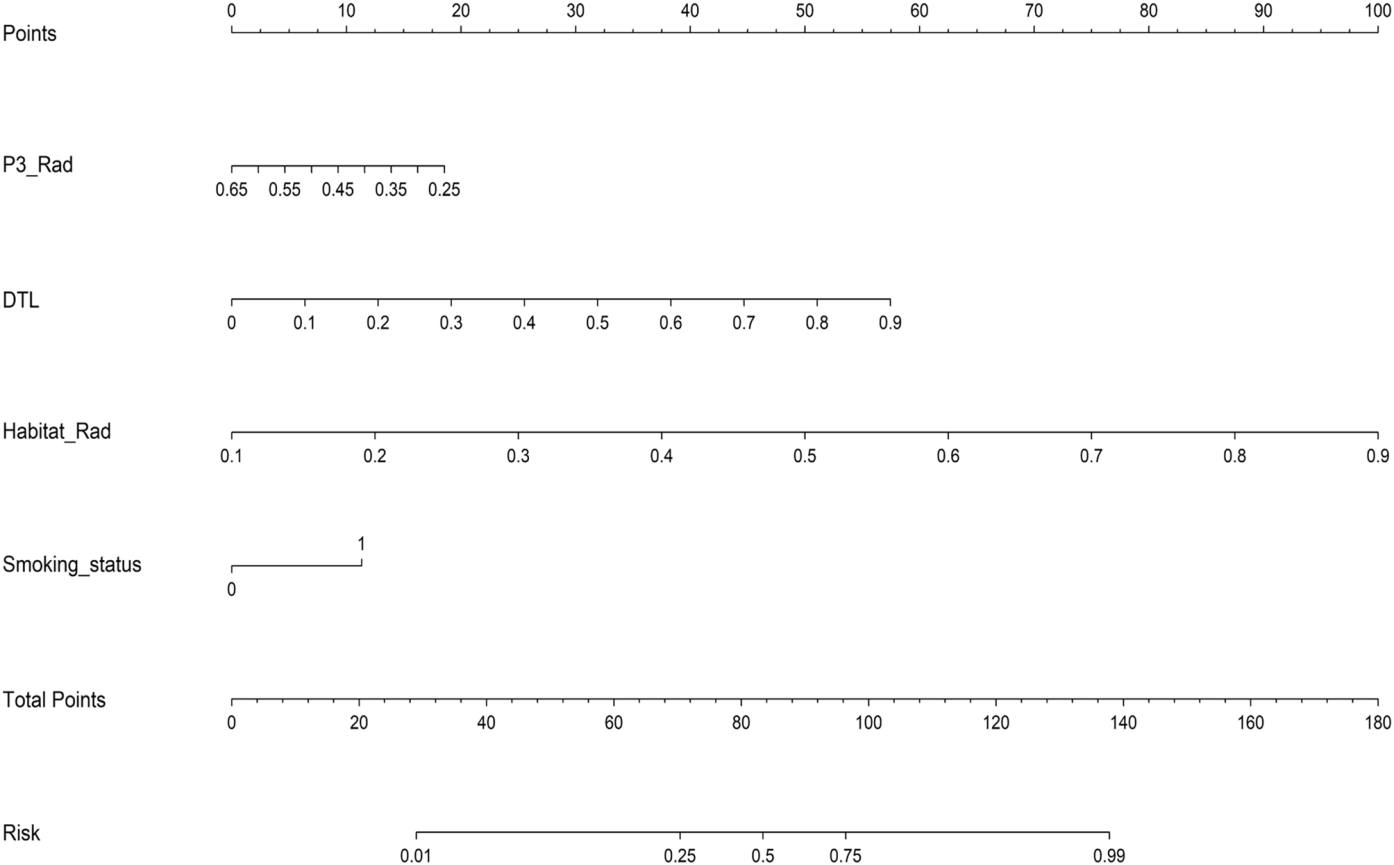
Shows the nomogram for clinical use.

We use the univariable analysis and stepwise multivariable analysis of clinical characteristics, Smoking status was identified as an independent factor associated with EGFR mutation status in the multivariate analysis and was therefore it was integrated with representative signatures (P3_Rad, DTL, Habitat_Rad) to create a nomogram (Fig. 5).

### Comparison of the performance of different models

**Figure 6:**
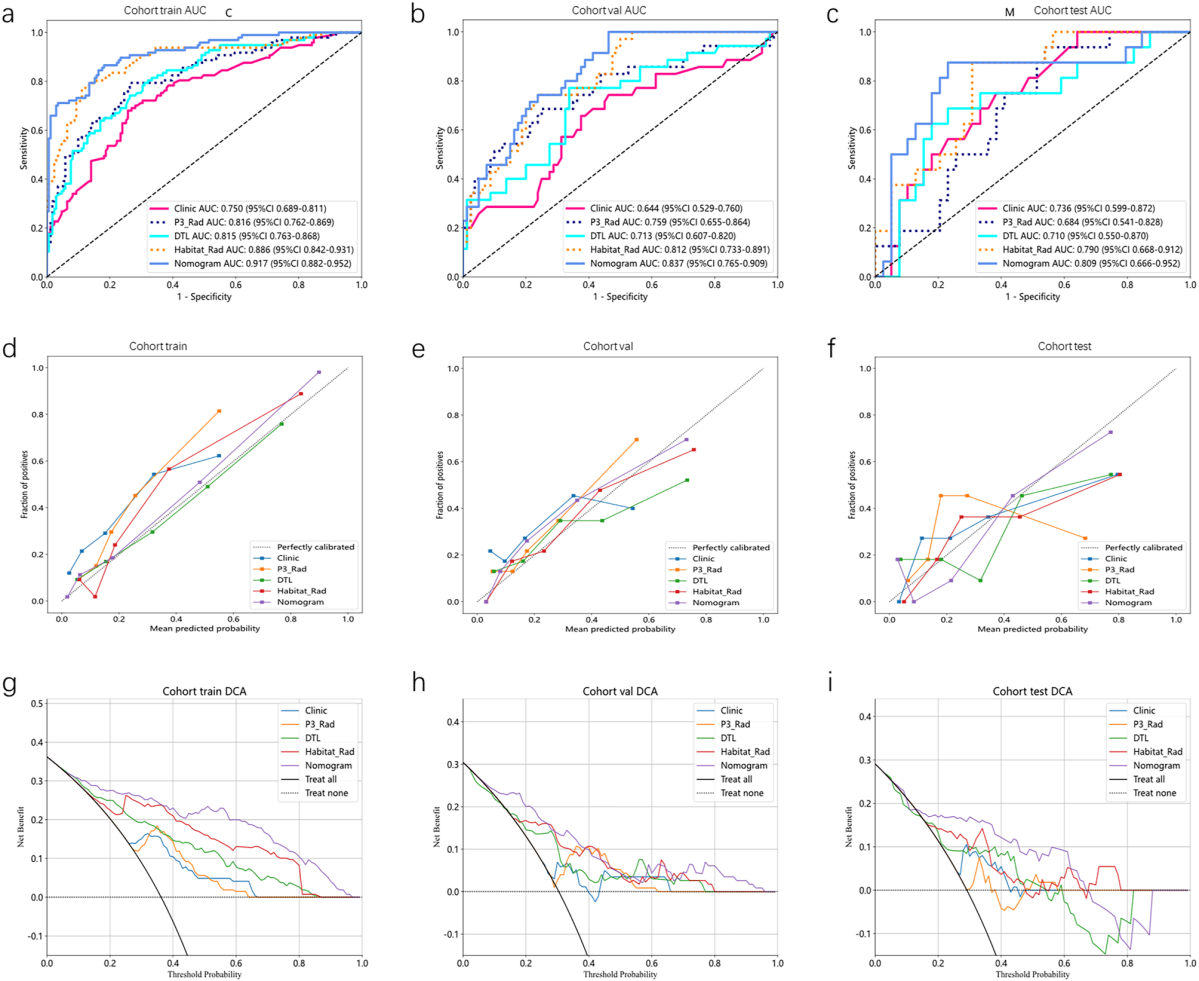
ROC curve comparison of different models (**a**), (**b**), (**c**) corresponding to the training, validation and external test sets, respectively. Calibration curve comparison of different models: (**d**), (**e**), (**f**) corresponding to the training, validation and external test sets, respectively. DCA curve comparison of different models: (**g**), (**h**), (**i**) corresponding to the training, validation and external test sets, respectively.

We compared the AUC values of the best models based on the above results for a more intuitive performance comparison (Fig. 6). In the train cohort, several signatures showed strong AUC values, with the highest AUC observed for the Nomogram signature (0.917, 95% CI: 0.882–0.952), closely followed by the Habitat_Rad signature (0.886, 95% CI: 0.842–0.931). The DTL signature also demonstrated a respectable AUC of 0.815 (95% CI: 0.763–0.868). In the validation cohort, the Nomogram signature continued to perform well with an AUC of 0.837 (95% CI: 0.765–0.909), maintaining its strength in distinguishing between classes. The Habitat_Rad and DTL signatures also exhibited competitive AUC values of 0.812 and 0.713 (95% CI: 0.733–0.891 and 0.607–0.820), respectively. In the external test cohort, the Nomogram signature maintained a strong AUC of 0.809 (95% CI: 0.666–0.952), accuracy of 0.800, sensitivity of 0.769, specificity of 0.875 (Supplementary Table S1).

The Hosmer–Lemeshow (HL) test was employed to construct a calibration curve. Compared to other signatures, our fusion model (Nomogram) yielded noticeable benefits based on the predicted probabilities. For further confirming the clinical gain of radiomic models, the decision curves were developed and compared in the five models, respectively. The nomogram proved to be the superior model due to its extensive range of thresholds in comparison to other models, resulting in superior net benefits across most threshold ranges. Evidence that a nomogram prediction model has the best clinical utility. Figure 6g–i correspond to the DCA curves of the training, validation, and external test sets, respectively.

## Discussion

This study introduces a comprehensive approach, encompassing intratumoral, peritumoral, habitat radiomics, and deep learning models, to predict EGFR mutation status in stage I NSCLC. The incorporation of habitat analysis and the development of a nomogram represent innovative contributions to the field. The findings underscore the potential of radiomics, particularly habitat analysis, in enhancing our understanding of tumor heterogeneity and predicting crucial molecular markers. The nomogram, integrating radiomic and clinical information, stands out as a valuable tool for personalized treatment planning in stage I NSCLC patients. Further research and validation are warranted to solidify the clinical applicability of these findings.

For Intra_Rad signatures, our present study has robust feature selection and high performance. Among the seven classifiers, the LightGBM classifier was found to offer the best effect with AUC of 0.821 (95% CI: 0.771–0.872), accuracy is 0.772 and sensitivities of 0.842. Our study demonstrated superior performance than some prior research^19–21^. However, they only concentrate on regions within the tumor, which overlooks the subtle changes in peritumoral microenvironments. Conversely, our study takes into consideration the potential impact of the peritumoral area. First, the peritumoral region may play a role in tumor invasion and metastasis, and it has been linked to prognosis^22,23^. Second, manual demarcation may have missed some tumor edge. A previous study^24^ that the AUC for peritumoral radiomics predicting EGFR mutations in early-stage NSCLC was mean 0.78 (range, 0.64–0.94). Our study shows improvement compared to theirs and have a multicenter patient population. We have used radiomic features to find that the peritumoral regions have a potential predictive ability for the prediction of the EGFR status, with the P3_Rad signature having the best performance. The AUC values of the training set, validation set and external test set in the peritumoral 3 mm region were 0.816, 0.759 and 0.684, respectively. This suggests that peritumoral radiomics is effective in predicting EGFR mutations.

Habitat analysis, also known as habitat imaging, is an imaging technique designed to capture subtle differences in tumors, and visualize spatial heterogeneity of cancer^25^. Gatenby et al.^26^ argues that cancer is not a single, self-organising system, but rather a patchwork of habitats, each subregion of the habitat imaging displays distinct environmental selection forces and cellular evolutionary strategies. Previous investigations^27,28^ supported the value of habitat radiomics in the diagnosis and prognosis of patients with lung cancer. While, the predictive ability of habitat analysis in determining EGFR mutation status in NSCLC remains uncertain. Our study conducted a habitat-based analysis and identified 13 features from each voxel. The model accurately predicted EGFR mutations with an AUC of 0.886 (95% CI: 0.842–0.931), an accuracy of 0.847 and a sensitivity of 0.889. The Habitat_Rad signature consistently exhibits the strongest discriminative power between different classes or conditions, as evidenced by its robust performance across all cohorts.

In contrast to radiomics, deep learning utilizes a nonlinear, hierarchical model structure inspired by the human brain's neural network to automatically extract features from input data without manual hard-coding^29^. During the study, three classic deep learning models were evaluated, with ResNet18 proving to be the most effective in terms of AUC (0.815, 95% CI: 0.763–0.868). This outperformed a previous study^30^ that reported an AUC of 0.738 for a deep learning model and 0.751 for a fusion model combining deep learning, imaging omics, and clinical features. Despite a smaller study population, our deep learning signature demonstrated better performance, encompassed multiple centers, and exhibited robustness across all cohorts.

The nomogram, incorporating multiple signatures, correctly predicted EGFR mutations with a high AUC of 0.917. Both the Nomogram and Habitat_Rad signatures consistently demonstrated excellent predictive ability across all cohorts. The nomogram provides a practical tool for doctors to assess the likelihood of EGFR mutation status based on relevant patient information, offering a valuable asset in clinical decision-making.

The present study has several limitations. First, the retrospective nature of the study introduces potential population selection bias, although efforts were made to enhance reliability through external validation. Second, the study focused solely on Asian populations, and the EGFR mutation profile may vary between ethnicities^31^. Further research is needed to determine the generalizability of the radiomics model to other regions or ethnic groups. Third, the study solely focused on EGFR mutation status and lacked assessments of patient efficacy and prognosis. Future research aims to delve into more comprehensive assessments, considering the potential of radiomics in evaluating the prognosis of stage I NSCLC patients^32^.

In conclusion, this study presents a novel and comprehensive approach, incorporating radiomics and deep learning models, to predict EGFR mutation status in stage I NSCLC. The nomogram, with its robust predictive ability, holds promise as a practical tool for clinicians. While acknowledging study limitations, these findings pave the way for further research and validation, emphasizing the potential of radiomics and deep learning in advancing personalized treatment strategies for NSCLC patients.

## Conclusion

In this study, a comprehensive analysis of CT image-based models was conducted to predict EGFR mutation status in stage I NSCLC patients. The habitat radiomic model emerged as superior to other models, showcasing its efficacy in capturing nuanced information from imaging data. The developed nomogram, integrating multiple radiomic models and smoking status, demonstrated feasibility and efficiency in predicting EGFR mutation status in stage I NSCLC patients. This non-invasive, cost-effective approach, encapsulated in the CT-based nomogram, holds promise as a valuable tool in guiding therapeutic decisions for the benefit of patients.

### Supplementary Information

Supplementary Information.

## Data Availability

The datasets used and/or analyzed during the current study available from the corresponding author on reasonable request.
