# Supplementary material for: Habitat radiomics and deep learning fusion nomogram to predict EGFR mutation status in stage I non-small cell lung cancer: a multicenter study
Source: Sci Rep. 2024 Jul 10;14:15877. doi: 10.1038/s41598-024-66751-1 (PMC11233600; doi:10.1038/s41598-024-66751-1)
Supplement: Supplementary file 1 — Supplementary Information. [file 41598_2024_66751_MOESM1_ESM.pdf]

Habitat radiomics and deep learning fusion nomogram to predict EGFR mutation status in stage I non-small cell lung cancer: a multicenter study

Jingran Wu[1#], Hao Meng[2#], Lin Zhou[3#], Meiling Wang[1], Shanxiu Jin[1], Hongjuan Ji[1], Bona Liu[1*], Peng Jin[4*] and Cheng Du[1*]

1. Department of Oncology, General Hospital of Northern Theater Command, Shenyang 110840, China.
2. Department of Thoracic Surgery, General Hospital of Northern Theater Command, Shenyang 110840, China.
3. Department of Thoracic Surgery, Yuebei People's Hospital Affiliated to Shantou University Medical College, Shaoguan 512025, China.
4. Department of Oncology, The Second Affiliated Hospital of Shandong First Medical University, Taian 271000, China.

# These authors have contributed equally to this work and share first authorship.
* These authors have contributed equally to this work and share corresponding authorship.

Corresponding Author:

1. Cheng Du, Department of Oncology, General Hospital of Northern Theater Command, Shenyang 110840, China. dc1115010@sina.com

2. Bona Liu, Department of Oncology, General Hospital of Northern Theater Command, Shenyang 110840, China. apbnaliu@sina.com

3. Peng Jin, Department of Oncology, The Second Affiliated Hospital of Shandong First Medical University, Taian 271000, China. 12196288@qq.com

Supplementary Data

Supplementary Data 1: Habitat Generation

1.1. ROI Local Feature Extraction

We employed a detailed process to extract local features from each voxel within the dataset using a $3 \times 3 \times 3$ moving window. This extraction captures a diverse array of measurements and attributes, such as intensity, texture, and other statistical properties, essential for delving into the intricate details of the dataset. These insights facilitate more precise modeling and analytical capabilities.

We extracted 13 radiomic features from each voxel, providing a multidimensional characterization of each subregion. These features include:

- **firstorder_Entropy**: Quantifies randomness or uncertainty in image values, calculated as entropy $= -\sum_{i=1}^{N_g} p(i)\log_2(p(i) + \epsilon)$.
- **firstorder_MeanAbsoluteDeviation (MAD)**: Represents the average distance of all intensity values from the image's mean value, defined as $\text{MAD} = \frac{1}{N_p}\sum_{i=1}^{N_p}|X(i) - \bar{X}|$.
- **Difference Entropy** and **Difference Variance**: Assess the variability and heterogeneity in neighborhood intensity values, respectively, with formulas difference_entropy $= \sum_{k=0}^{N_g-1} p_{x-y}(k)\log_2\big(p_{x-y}(k) + \epsilon\big)$ and difference_variance $= \sum_{k=0}^{N_g-1}(k - DA)^2 p_{x-y}(k)$.
- **Joint Energy** and **Joint Entropy**: Evaluate the homogeneity and randomness of intensity patterns in the image, given by joint_energy $= \sum_{i=1}^{N_g}\sum_{j=1}^{N_g}\big(p(i,j)\big)^2$ and joint_entropy $= -\sum_{i=1}^{N_g}\sum_{j=1}^{N_g} p(i,j)\log_2(p(i,j) + \epsilon)$.
- Other features like firstorder_Median, glcm_DifferenceAverage, glcm_DifferenceEntropy, glcm_DifferenceVariance, glcm_Imc1, glcm_Imc2 details in http://pyradiomics.readthedocs.io

1.2. Details of Kmeans for Subregion Clustering

The K-means algorithm was utilized to analyze the multidimensional feature space derived from the radiomic features. This clustering method grouped all voxels and their associated characteristics into distinct clusters, exploring a range of cluster centers from 3 to 10 to categorize unique habitat regions within the ROI. The effectiveness of the clustering was evaluated using the Calinski-Harabasz score, which aided in selecting the optimal number of clusters.

The K-means algorithm partitions data into K distinct clusters, iteratively updating the centroids of these clusters to minimize the within-cluster sum of squares. The objective function of the K-means algorithm, which is crucial for effective clustering, is defined as follows:

$$J = \sum_{i=1}^{N} \sum_{k=1}^{K} w_{ik} \times \| x_i - \mu_k \|^2$$

Where:

- J represents the objective function.
- N denotes the number of data points.
- K is the number of clusters.
- w_{ik} is a binary indicator (1 if data point i is in cluster k, 0 otherwise).
- x_i is the ith data point.
- μ_k is the centroid of cluster k.
- $\| x_i - \mu_k \|^2$ is the squared Euclidean distance between data point i and centroid k.

Habitat Region Synthesis: After clustering, subregions sharing the same cluster IDs were merged to form comprehensive habitat regions, each representing distinct microenvironmental characteristics within the ROI.

Supplementary Data 2: Optimized hyper-parameters for each machine learning model

For the Logistic Regression model, we used no penalty and set the maximum number of iterations to 100. The Random Forest model was configured with 25 estimators, a maximum depth of 4, and a minimum sample split of 4. Our XGBoost model utilized 20 estimators with a binary logistic objective, a maximum depth of 2, and a minimum child weight of 1.2, while not using the label encoder and setting the evaluation metric to error. The LightGBM model was set up with 15 estimators, a maximum depth of 3, and a minimum child weight of 0.5. Lastly, the Extra Trees model was tuned with 40 estimators, a maximum depth of 3, and a minimum sample split of 2.

Supplementary Figures

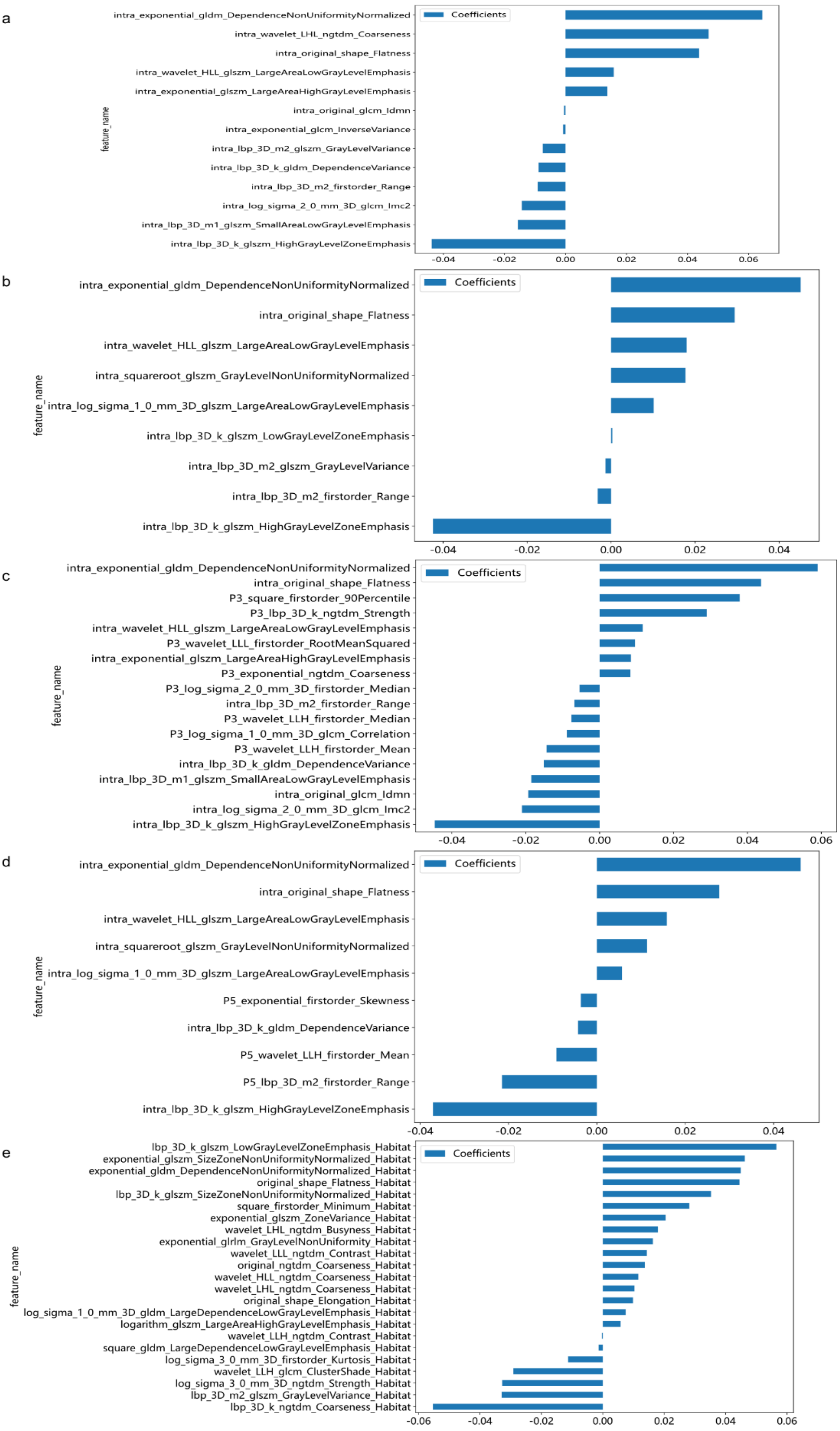

a
feature_name
Coefficients
intra_exponential_gldm_DependenceNonUniformityNormalized
intra_wavelet_LHL_ngtdm_Coarseness
intra_original_shape_Flatness
intra_wavelet_HLL_glszm_LargeAreaLowGrayLevelEmphasis
intra_exponential_glszm_LargeAreaHighGrayLevelEmphasis
intra_original_glcm_Idmn
intra_exponential_glcm_InverseVariance
intra_lbp_3D_m2_glszm_GrayLevelVariance
intra_lbp_3D_k_gldm_DependenceVariance
intra_lbp_3D_m2_firstorder_Range
intra_log_sigma_2_0_mm_3D_glcm_Imc2
intra_lbp_3D_m1_glszm_SmallAreaLowGrayLevelEmphasis
intra_lbp_3D_k_glszm_HighGrayLevelZoneEmphasis
-0.04 -0.02 0.00 0.02 0.04 0.06
b
feature_name
Coefficients
intra_exponential_gldm_DependenceNonUniformityNormalized
intra_original_shape_Flatness
intra_wavelet_HLL_glszm_LargeAreaLowGrayLevelEmphasis
intra_squareroot_glszm_GrayLevelNonUniformityNormalized
intra_log_sigma_1_0_mm_3D_glszm_LargeAreaLowGrayLevelEmphasis
intra_lbp_3D_k_glszm_LowGrayLevelZoneEmphasis
intra_lbp_3D_m2_glszm_GrayLevelVariance
intra_lbp_3D_m2_firstorder_Range
intra_lbp_3D_k_glszm_HighGrayLevelZoneEmphasis
-0.04 -0.02 0.00 0.02 0.04
c
feature_name
Coefficients
intra_exponential_gldm_DependenceNonUniformityNormalized
intra_original_shape_Flatness
P3_square_firstorder_90Percentile
P3_lbp_3D_k_ngtdm_Strength
intra_wavelet_HLL_glszm_LargeAreaLowGrayLevelEmphasis
P3_wavelet_LLL_firstorder_RootMeanSquared
intra_exponential_glszm_LargeAreaHighGrayLevelEmphasis
P3_exponential_ngtdm_Coarseness
P3_log_sigma_2_0_mm_3D_firstorder_Median
intra_lbp_3D_m2_firstorder_Range
P3_wavelet_LLH_firstorder_Median
P3_log_sigma_1_0_mm_3D_glcm_Correlation
P3_wavelet_LLH_firstorder_Mean
intra_lbp_3D_k_gldm_DependenceVariance
intra_lbp_3D_m1_glszm_SmallAreaLowGrayLevelEmphasis
intra_original_glcm_Idmn
intra_log_sigma_2_0_mm_3D_glcm_Imc2
intra_lbp_3D_k_glszm_HighGrayLevelZoneEmphasis
-0.04 -0.02 0.00 0.02 0.04 0.06
d
feature_name
Coefficients
intra_exponential_gldm_DependenceNonUniformityNormalized
intra_original_shape_Flatness
intra_wavelet_HLL_glszm_LargeAreaLowGrayLevelEmphasis
intra_squareroot_glszm_GrayLevelNonUniformityNormalized
intra_log_sigma_1_0_mm_3D_glszm_LargeAreaLowGrayLevelEmphasis
P5_exponential_firstorder_Skewness
intra_lbp_3D_k_gldm_DependenceVariance
P5_wavelet_LLH_firstorder_Mean
P5_lbp_3D_m2_firstorder_Range
intra_lbp_3D_k_glszm_HighGrayLevelZoneEmphasis
-0.04 -0.02 0.00 0.02 0.04
e
feature_name
Coefficients
lbp_3D_k_glszm_LowGrayLevelZoneEmphasis_Habitat
exponential_glszm_SizeZoneNonUniformityNormalized_Habitat
exponential_gldm_DependenceNonUniformityNormalized_Habitat
original_shape_Flatness_Habitat
lbp_3D_k_glszm_SizeZoneNonUniformityNormalized_Habitat
square_firstorder_Minimum_Habitat
exponential_glszm_ZoneVariance_Habitat
wavelet_LHL_ngtdm_Busyness_Habitat
exponential_glrlm_GrayLevelNonUniformity_Habitat
wavelet_LLL_ngtdm_Contrast_Habitat
original_ngtdm_Coarseness_Habitat
wavelet_HLL_ngtdm_Coarseness_Habitat
wavelet_LHL_ngtdm_Coarseness_Habitat
original_shape_Elongation_Habitat
log_sigma_1_0_mm_3D_gldm_LargeDependenceLowGrayLevelEmphasis_Habitat
logarithm_glszm_LargeAreaHighGrayLevelEmphasis_Habitat
wavelet_LLH_ngtdm_Contrast_Habitat
square_gldm_LargeDependenceLowGrayLevelEmphasis_Habitat
log_sigma_3_0_mm_3D_firstorder_Kurtosis_Habitat
wavelet_LLH_glcm_ClusterShade_Habitat
log_sigma_3_0_mm_3D_ngtdm_Strength_Habitat
lbp_3D_m2_glszm_GrayLevelVariance_Habitat
lbp_3D_k_ngtdm_Coarseness_Habitat
-0.06 -0.04 -0.02 0.00 0.02 0.04 0.06

Fig S1. The histogram of the coefficients of the selected features. (a) 13 optimal features selected from the intratumoral region; (b), (c), (d) 9, 18, 10 optimal features were selected from the Peritumoral 1mm, 3mm and 5mm regions; (e) 23 optimal features selected from the habitat region.

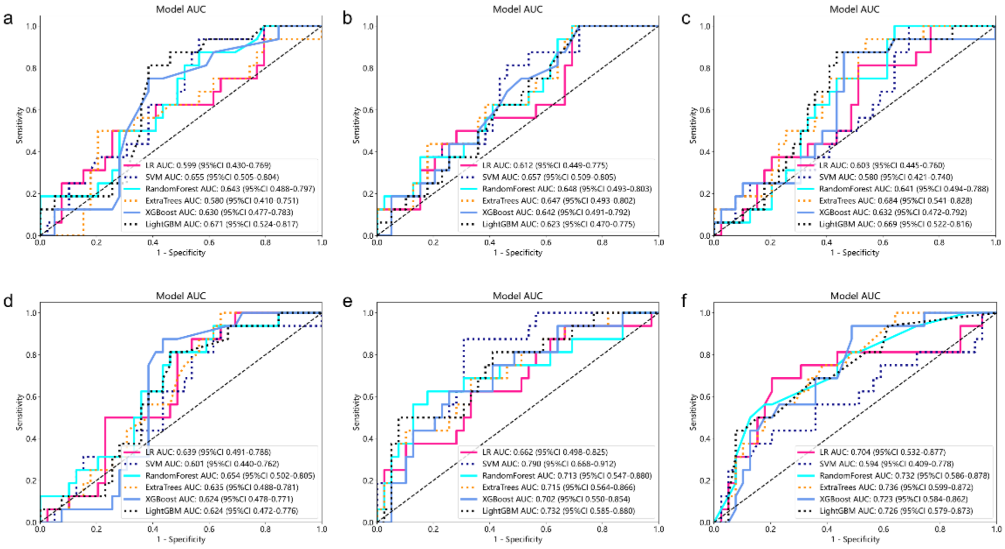

Fig S2. Comparison of ROC curves for different machine learning methods on the external test set. (a) ROC curve comparison of different machine learning models for intratumoral radiomics signature; (b) (c), (d) are ROC curve comparisons of different machine learning models for peritumoral 1 mm, 3 mm and 5 mm radiomics signatures, respectively. (e) ROC curve comparison of different machine learning models for habitat radiomics signature. (f) ROC curve comparison of different machine learning models for clinical signature.

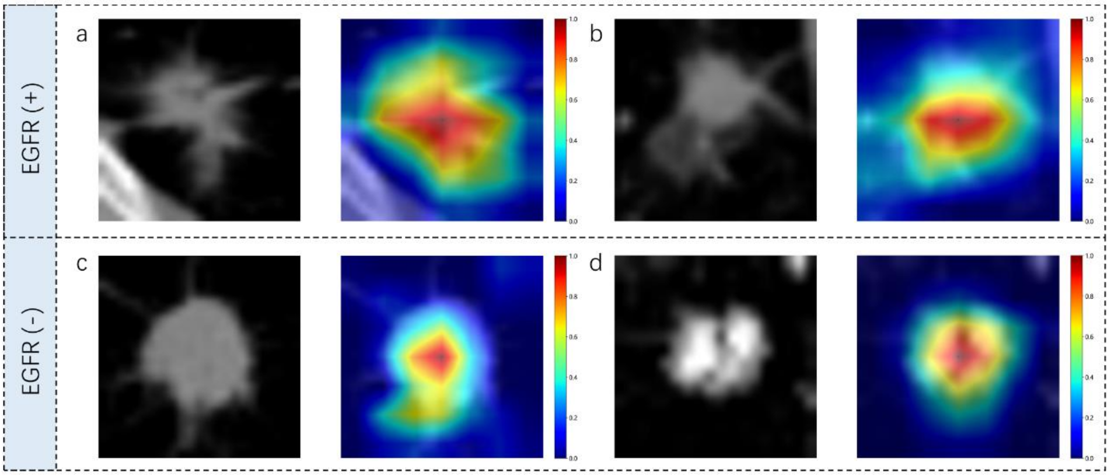

Fig S3. Gradient-weighted Class Activation Mapping (Grad-CAM) of four different patients. (a, b) two patients with EGFR mutant type. (c, d) two patients with EGFR wild type.

Supplementary Table

Table S1. The performance comparison of different signatures.

Signature	cohort	AUC（95% CI）	Accuracy	Sensitivity	Specificity	PPV	NPV
Intra_Rad	Train	0.821（0.771 - 0.872）	0.772	0.842	0.649	0.809	0.700
	Validation	0.746（0.633 - 0.858）	0.774	0.850	0.656	0.829	0.636
	Test	0.671（0.524 - 0.817）	0.673	0.615	0.812	0.889	0.464
P1_Rad	Train	0.811（0.755 - 0.866）	0.757	0.743	0.784	0.858	0.633
	Validation	0.754（0.648 - 0.861）	0.800	0.900	0.571	0.828	0.714
	Test	0.657（0.509 - 0.805）	0.618	0.538	0.812	0.875	0.419
P3_Rad	Train	0.816（0.762 - 0.870）	0.754	0.731	0.794	0.862	0.626
	Validation	0.759（0.655 - 0.864）	0.730	0.750	0.686	0.845	0.545
	Test	0.684（0.541 - 0.828）	0.600	0.462	0.938	0.947	0.417
P5_Rad	Train	0.758（0.699 - 0.816）	0.642	0.532	0.835	0.850	0.503
	val	0.719（0.609 - 0.830）	0.748	0.850	0.514	0.800	0.600
	Test	0.635（0.488 - 0.781）	0.545	0.359	1.000	1.000	0.390
Habitat_Rad	Train	0.886（0.842 - 0.931）	0.847	0.889	0.773	0.874	0.798
	Validation	0.812（0.733 - 0.891）	0.757	0.762	0.743	0.871	0.578
	Test	0.790（0.668 - 0.912）	0.745	0.692	0.875	0.931	0.538
Clinical	Train	0.750（0.689 - 0.811）	0.720	0.743	0.680	0.804	0.600
	Validation	0.644（0.529 - 0.760）	0.635	0.625	0.657	0.806	0.434
	Test	0.736（0.599 - 0.872）	0.655	0.615	0.750	0.857	0.444
Nomogram	Train	0.917（0.882 - 0.952）	0.836	0.819	0.866	0.915	0.730
	Validation	0.837（0.765 - 0.909）	0.678	0.537	1	1	0.486
	Test	0.809（0.666 - 0.952）	0.800	0.769	0.875	0.937	0.609

Intra_Rad, intratumoral radiomics signature; P1_Rad, Peritumoral 1 mm radiomics signature; P3_Rad, Peritumoral 3mm radiomics signature; P5_Rad, Peritumoral 5mm radiomics signature; Habitat_Rad, habitat radiomics signature. PPV, positive predictive value; NPV, negative predictive value.
